# Cost-effectiveness of different human papillomavirus vaccines in Singapore

**DOI:** 10.1186/1471-2458-11-203

**Published:** 2011-03-31

**Authors:** Vernon J Lee, Sun Kuie Tay, Yee Leong Teoh, Mei Yin Tok

**Affiliations:** 1 Center for Health Services Research, National University of Singapore, Singapore; 2 Department of Epidemiology and Public Health, National University of Singapore, Singapore; 3 Department of Obstetrics & Gynaecology, Singapore General Hospital, Singapore; 4 GlaxoSmithKline Biologicals, Singapore

**Keywords:** Cervical cancer, cervical intraepithelial neoplasia, incremental cost effectiveness ratio, vaccine

## Abstract

**Background:**

Human papillomavirus (HPV) vaccines are widely available and there have been studies exploring their potential clinical impact and cost-effectiveness. However, few studies have compared the cost-effectiveness among the 2 main vaccines available - a bivalent vaccine against HPV 16/18, and a quadrivalent vaccine against 6/11/16/18. We explore the cost-effectiveness of these two HPV vaccines in tropical Singapore.

**Methods:**

We developed a Markov state-transition model to represent the natural history of cervical cancer to predict HPV infection, cancer incidence, mortality, and costs. Cytologic screening and treatment of different outcomes of HPV infection were incorporated. Vaccination was provided to a cohort of 12-year old females in Singapore, followed up until death. Based on available vaccines on the market, the bivalent vaccine had increased effectiveness against a wider range of HPV types, while the quadrivalent vaccine had effectiveness against genital warts. Incremental cost-effectiveness ratios (ICER) compared vaccination to no-vaccination, and between the two vaccines. Sensitivity analyses explored differences in vaccine effectiveness and uptake, and other key input parameters.

**Results:**

For the no vaccination scenario, 229 cervical cancer cases occurred over the cohort's lifetime. The total discounted cost per individual due to HPV infection was SGD$275 with 28.54 discounted life-years. With 100% vaccine coverage, the quadrivalent vaccine reduced cancers by 176, and had an ICER of SGD$12,866 per life-year saved. For the bivalent vaccine, 197 cancers were prevented with an ICER of $12,827 per life-year saved. Comparing the bivalent to the quadrivalent vaccine, the ICER was $12,488 per life-year saved. However, the cost per QALY saved for the quadrivalent vaccine compared to no vaccine was $9,071, while it was $10,392 for the bivalent vaccine, with the quadrivalent vaccine dominating the bivalent vaccine due to the additional QALY effect from reduction in genital warts. The overall outcomes were most sensitive to vaccine cost and coverage.

**Conclusion:**

HPV vaccination is a cost-effective strategy, and should be considered a possible strategy to reduce the impact of HPV infection.

## Background

Cervical cancer is the second most common cancer in women worldwide [1], affecting 500,000 women annually and resulting in more than 250,000 deaths [2]. In Singapore, despite decreasing incidence of cervical cancer as a result of regular cytological screening, it remains a common gynaecological cancer [3], with 200 cases and about 100 deaths annually [3,4].

Human papillomavirus (HPV) infection is the main cause of cervical cancer. There are more than 150 different strains of HPV but only about 15 are high-risk oncogenic strains for cervical cancer. Of these, HPV types 16 and 18 account for about 70% of all cervical cancer cases, while the other oncogenic strains account for the rest [5-7]. Two prophylactic HPV vaccines, a bivalent vaccine which targets HPV 16/18, and a quadrivalent vaccine which targets 6/11/16/18, are widely available. Both vaccines have shown to be highly effective in clinical trials and economic studies of HPV vaccines have found them to be cost-effective in various countries [8]. As such, many countries are considering universal vaccination of women with the HPV vaccine.

Before any universal vaccine implementation, it is important for policymakers to understand the long-term benefits (beyond the time horizon of clinical trials) of the vaccine by using mathematical modelling in a decision-analytic framework [9-17]. Extending previous studies of HPV vaccination, we compare the status quo of cervical cancer screening only and vaccination with a quadrivalent or bivalent vaccine in addition to baseline screening. This allowed us to determine the cost-effectiveness of vaccination over the current screening practices, and the incremental cost-effectiveness of one vaccine over the other.

## Methods

**Figure 1:**
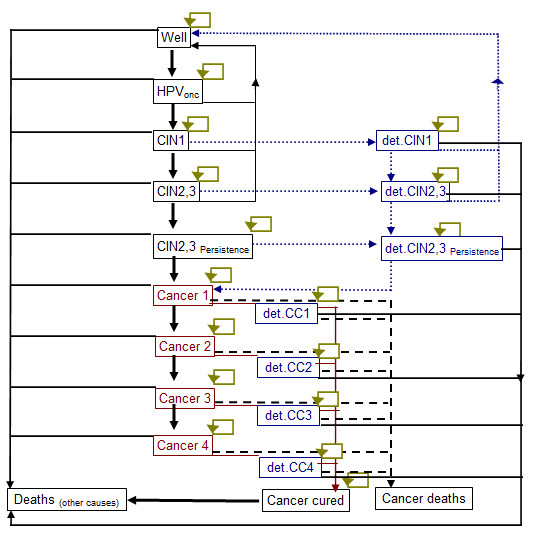
Markov model for the history of cervical cancer

We developed a deterministic Markov state-transition model based on the natural history of HPV infection and cervical cancer in the tropical South-East Asian city-state Singapore (Figures 1). We assumed that the natural history is relatively long, therefore the cycles were annual [18,19]. The model was performed on a hypothetical cohort of women in Singapore who turned 12 years old in 2008, which is the typical age of HPV vaccination. This amounted to a mid-year female population of 25,000 [20] and the model followed this cohort until death - 88 cycles, assumed to correspond to the time needed until everyone has died. We assumed that sexual activity and hence HPV infection only occurred after age 12.

Life expectancies and annual mortality rates for the cohort were obtained from the Singapore Department of Statistics [20]. For the input variables (Table 1) [4,18-29], we obtained data from local sources where available, including disease incidence and costs of interventions and outcomes. Most studies on disease evolution and transition between different disease states were performed by a few large international studies - we therefore relied on these to obtain input parameters. The variables were calibrated to the local setting based on local incidence data.

**Table 1:** Vaccination, screening, cost parameters and transition probabilities

Parameters	Base case	Ranges	Source
**Vaccination**			
Cohort size	25,000		[20]
Vaccine coverage	100%		Assumption
Age at vaccination	12		
Vaccine efficacy* - bivalent vaccine	87.2%	70%-95%	[21,22]
Vaccine efficacy* - quadrivalent vaccine	78.8%	70%-95%	[23]
Duration of efficacy	Lifetime	5 years to lifetime	Assumption
Vaccine waning + booster	None	5, 10, 20 years	Assumption
			
**Screening**			
Screening age range	25-65		[4]
Screening interval	every 3 years		[4]
Percentage screened per year	17%	10-20	Estimated
Cytology sensitivity to detect CIN1	0.58	none	[18,19]
Cytology sensitivity to detect CIN2/3	0.61	none	[18,19]
Compliance to CIN1 treatment	75%	50%-100%	Assumption
Compliance to CIN2/3 treatment	75%	50%-100%	Assumption
			
**Cancer detection rate**	*Low*	*High*	
Probability Of symptons CC1	0.075	0.185	
Probability Of symptons CC2	0.113	0.3	
Probability Of symptons CC3	0.3	0.75	[24,25]
Probability Of symptons CC4	0.45	0.8	
			
**Cost, SGD$ (2008)**		*(-20%/+50%) except vaccine cost (200-400)*	
Vaccine cost per vaccinated woman, bivalent vaccine #	400		
Vaccine cost per vaccinated woman, quadrivalent vaccine#	400		Assumption
Cytology test^	40		
Colposcopy and Biopsy^	207		
CIN 1 treatment	1,105		
CIN 2/3 treatment	1,480		
Stage 1 Cancer treatment cost	9,388		Singapore public sector hospitals
Stage 2 Cancer treatment cost	9,765		
Stage 3 Cancer treatment cost	9,765		
Stage 4 Cancer treatment cost	11,047		
Genital warts	750		
			
**Discounting**			
Costs, Outcomes	3%, 3%	(0%-5%)	Assumption
			
**Transition Probabilities**			
Well to HPV	0.05	0-0.2	
HPV to clearance	0.4	0.29-0.55	
HPV to CIN1	0.05	0.014-0.14	[18,19,26]
CIN1 clearance	0.4	0.24-0.5	
CIN1 to CIN2/3	0.09	0.02-0.32	
CIN2/3 clearance	0.25	0.01-0.45	
CIN2/3 to persistent CIN2/3	0.11	0.03-0.20	
Persistent CIN2/3 to Cancer stage 1	0.05	0.001-0.15	
Cancer stage 1 to Cancer stage 2	0.22	0.11-0.4	
Cancer 1 to cancer cured	0.84	0.63-0.98	
Cancer stage 1 to Cancer stage 3	0.24	0.12-0.5	
Cancer 2 to cancer cured	0.66	0.49-0.83	
Cancer stage 3 to Cancer stage 4	0.24	0.12-0.8	
Cancer 3 to cancer cured	0.38	0.28-0.48	[21,22,24-26]
Cancer 4 to cancer cured	0.11	0.08-0.14	
Detected CIN1 to well	0.9	0.8-1	
Detected CIN1 to Detected CIN2/3	0.09	0.02-0.32	
Detected CIN2/3 to Well	0.9	0.8-1	
Detected CIN2/3 to Detected PCIN2/3	0.11	0.03-0.20	
Detected PCIN2/3 to Cancer 1	0.05	0.001-0.15	
Mortality of cervical cancer	0.11		
			
**Cervical Warts**			
Effectiveness of vaccination against warts	0.90		[27,28]
Cost of treatment	750		
Well to low risk HPV	0.050		
Low risk HPV to well	0.500		
Low risk HPV to CIN1 low risk	0.036		
Low risk HPV to warts	0.027		
Warts to well	0.875		
CIN1 low risk to well	0.500		
CIN1 low risk to detected CIN 1 low risk	0.099		
Detected CIN 1 low risk to well	0.950		
			
**QALYs**			
Disease free	1		
Genital warts	0.96	0.91-0.99	[10,25,29]
Detected CIN	0.89	0.84-0.94	
Cancer detected			
Stage I	0.65	0.49-0.81	
Stage II	0.56	0.42-0.70	
Stage III	0.56	0.42-0.70	
Stage IV	0.48	0.36-0.60	
Cancer cured	0.94		

*Calculated, refer to Table 1b.

^#^including costs of implementing, administration and support for vaccination programme

^including admininistrative cost & patient costs

### Transition probabilities

HPV infection progresses to cervical intraepithelial neoplasia (CIN), the precursor of cervical cancer. The usual progression is from CIN 1 to CIN 2, CIN 3, and persistent forms of the latter 2 CIN stages. CIN 2,3 is grouped together due to similar outcomes and management, while the persistent forms are direct precursors of cervical cancer. Cervical cancers are divided into 4 prognostic stages 1 to 4. Each progressive stage is associated with poorer prognosis, lower treatment success rates, and higher recurrence and relapse rates. The transition probabilities for disease progression were obtained from previously published sources (Table 1). To account for uncertainty, we performed wide sensitivity analyses.

### Screening

In Singapore, recommended screening for cervical cancer starts at age 25 years for women who have ever had sexual intercourse and at 3 yearly intervals if previous smears were negative, until the age of 69 if all smears are negative. In 2004, a survey showed that across all age groups, only 52% of women had their Pap smear done in the last 3 years, but most women did not undergo regular screening every 3 years [4]. We therefore assumed that Singaporean women would on average start screening at 25 years of age, and 17% of the cohort would undergo screening each year.

We assumed that the sensitivity of PAP smears were 58% for CIN 1 and 61% for CIN2,3 [30,31]. If a smear is abnormal, further tests including colposcopy and if necessary biopsy are introduced as per local abnormal pap smear guidelines [41], and treatment performed as clinically indicated. We also assumed that once positive, all CIN states would be confirmed by colposcopy and biopsy, and 75% of CIN1 and 75% of CIN2, 3 cases would comply to treatment with an efficacy of 90% [32].

### Treatment

For CIN, the chance of cure is almost 100% and we assumed a mean cost of treatment for the various CIN stages. For cervical cancer, the chance of cure depends on the cancer stage, while the treatment cost depends on the treatment type. We also determined the chance of symptoms occurring which would lead to early detection and treatment. We modeled these outcomes and costs based on local data obtained from hospitals. As local data on survival rates together with disease transition probabilities were not available, and overall survival rates in Singapore were comparable to developed countries [42], we used available cancer survival rates from similar developed countries [21,22]. These survival rates were annualized across 5 years to determine overall cure rates for each year - we assumed that those who survive beyond 5 years were cured. Once cancer was detected, we assumed that all individuals would undergo treatment. We did not model outcomes of treatment as individual state transitions but attributed an overall cost to each state.

### Vaccination

In Singapore, the quadrivalent and bivalent vaccines have been licensed since 2006 and 2007 respectively. Vaccination remains voluntary, and privately funded individually. To compare between the vaccines, we assumed that the bivalent vaccine has a higher efficacy against other non-16/18 high-risk HPV types compared with the quadrivalent vaccine [32] using a technique described by Debicki et al [25] and shown in Table 2. However, the quadrivalent vaccine has additional protection against other low-risk HPV-types that cause genital warts and CIN 1 - the bivalent vaccine is assumed to have no such protection.

**Table 2:** Details of efficacy calculations

			Source
	**Bivalent** ** vaccine**	**Quadrivalent** ** vaccine**	

**Reduction in the probability of HPV infection**			
Assumed proportion of HPV 16/18 in cervical cancer, A	74.9%	74.9%	[7]
Vaccine efficacy-percent reduction in HPV 16/18 persistent infections, B	95.0%	95.0%	[23,27,28,32-40]
Assumed proportion of other high risk HPV in cervical cancer, C	23.4%	23.4%	[7]
Vaccine efficacy-percent reduction in other high risk HPV persistent infections, D	68.4%	32.5%	[30-32]
Calculated reduction in the probability of HPV infection (AxB)+(CxD)	87.2%	78.8%	
			
**Corection factor for CIN1**			
Percent of HPV 1618 in CIN1 cases which are caused by oncogenic HPV, E	37.0%		
Correction factor for CIN1, A-E	37.9%		
			
**Corection factor for CIN2/3**			
Percent of HPV 1618 in CIN2/3 cases which are caused by oncogenic HPV, F	52.0%		
Correction factor for CIN2/3 A-F	22.9%		

Based on recent studies, the range of protection for non-16/18 oncogenic HPV types are between 53.0% and 68.2% for the bivalent vaccine [23,33] and 32.5% for the quadrivalent vaccine [21]. The overall effectiveness for the quadrivalent vaccine has been shown to be about 75% [34]. In addition, the quadrivalent vaccine has a 90% protection against HPV-types that cause genital warts [27,28]

Base case vaccine characteristics are assumed as follows: (1) proportion of individuals protected following immunization is 100%; (2) vaccine duration is life-long; (3) effectiveness of both vaccines against HPV types 16/18 are similar at 95% [23,33-40]; (4) to create fair competition, we assumed that the prices of both vaccines were equivalent and includes all vaccination costs; (5) we did not include therapeutic benefits to vaccinated patients already infected and assumed that the natural disease history is unaltered; (6) we assumed that all girls based on the coverage rate would receive the full vaccine course and be immunized after 1 year. We performed sensitivity analyses on these assumptions.

### CIN State Adjustments

For the CIN states, we had to determine the actual proportion of CIN cases that are reduced by vaccination, as not all CIN states are affected by vaccination as compared to cancer. This is to allow for the reduction proportion to be accurate as this will influence the costs of the different strategies. We used a correction method well described by Debicki et al [25] where we determined the proportion of CIN 1 and CIN2/3 cases that were caused by non-oncogenic viruses in the no vaccine strategy, and added these numbers to those obtained for oncogenic viruses in the vaccination strategy (Table 2).

### Genital Warts

Genital warts are another manifestation of HPV infection caused by non-oncogenic HPV viruses. Low-oncogenic-risk HPV-6/11 is responsible for >95% percent of genital warts [43,44]. Genital warts cause superficial symptoms but do not result in cancer, and are therefore a separate outcome of HPV infection.

**Figure 2:**
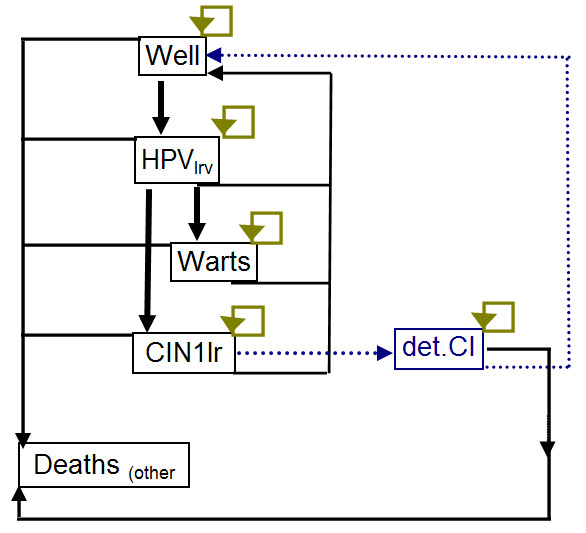
Markov model for the history of genital warts

The model for the development of genital warts is shown in Figure 2. We assumed that low-oncogenic-risk HPV types can either cause clinical genital warts or CIN 1. Clinical genital warts would be identified clinically and treated. CIN 1 would be detected through the normal screening process and we assumed would be indistinguishable from CIN 1 caused by oncogenic types. A reduction in CIN 1 caused by low-oncogenic-risk HPV types would contribute to the overall reduction in CIN 1 incidence [45].

### Costs, Utility, and discounting

Costs of the various interventions and outcomes were obtained from patients with HPV infection, CIN states, and cervical cancer diagnosed in 2004 across public sector hospitals in Singapore (SK Tay, unpublished data). All medical services were traced and costs collected from hospital finance data for the following 5 years or until death, and averaged across all patients. We performed the analysis starting from the year 2008 and all interventions according to technology available in 2008. We adopted the health services perspective and included all direct medical costs and benefits. Vaccination cost includes the vaccine cost and related administrative fees. All costs are represented in 2008 Singapore dollars (2008 exchange rate, USD1: SGD1.416).

For the Quality-Adjusted Life Years (QALYs) calculations, the utility values were obtained from other developed countries as similar data was not available locally (Table 1a). Utility for each health state, which ranged from 1 which corresponded to complete health to 0 for death, were multiplied by the time spent in each state. Genital warts and detected CIN (colposcopy treatment) was given utility values of 0.96 and 0.89 respectively [29]. Quality values for various detected cancer stages differed depending on the cancer stage, while cured cancer was slightly less than complete health [10,25].

Since the model ran over a long time-frame, costs and benefits were translated into present values using a discount factor [46-49]. Health outcomes and costs were discounted at 3% per annum, similar to previous local economic evaluations.

### Analyses

The model was run using Excel spreadsheets (Microsoft Corp, Redmond, WA) and @Risk (Palisade, Newfield, NY) simulation add-in. We performed cost-benefit analysis using the costs per life-year saved with individual economic value calculated from the net present value of future annual earnings (earnings-equivalent for the elderly), adjusted for age [50]. We also performed cost-effectiveness analyses; and cost-utility analyses as not all negative health outcomes resulted in death. The incremental cost-effectiveness ratio (ICER) would be acceptable if it is below the per-capita gross domestic product (GDP) for the population. The per-capita GDP for Singapore in 2008 was $53,192.

All parameters were subject to sensitivity analysis to determine which input variables were most important in determining the final strategy. We performed one-way and two-way sensitivity analyses on vaccination and transition parameters to determine the impact of changes in input variables. This allowed us to determine which parameters had the highest influence on the outcome, and would be priority areas for intervention.

## Results

### Base case analysis

Table 3 reports the base-case results of estimated cases, non-discounted and discounted total costs, life-years, and QALYs. The model estimates that approximately 86% of cervical cancer, 86% of deaths from cancer, and 47% of CIN 1, 65% of CIN 2/3, and 86% of persistent CIN 2/3 cases could be prevented by the bivalent vaccine vaccination. The quadrivalent vaccine avoids these cases by 2-9% less than the bivalent vaccine. However, the quadrivalent vaccine reduces genital warts cases by 89%. Vaccination with the bivalent or quadrivalent vaccine yielded 634 and 570 respectively more life years than screening alone.

**Table 3:** Summary of estimated cases, non-discounted and discounted total costs and life-years of a single age-cohort (n = 25,000) of 12 year old girls

		Screening only	Bivalent*	Quadrivalent*
Cases				
	Cervical cancer	229	32	53
	Deaths from cervical cancer	144	20	33
	CIN Persistent 2n3 detected	2,073	294	480
	CIN 2n3 detected	382	135	168
	CIN 1 detected	2,109	1,127	1,293
	Genital warts	4,126	4,126	447
				
NON-DISCOUNTED				
Total costs		21,150,496	25,020,340	22,822,396
	Vaccine costs	-	10,000,000	10,000,000
	Screening costs	11,006,014	9,949,324	10,078,234
	CIN1 treatment costs	2,330,522	1,245,203	1,292,796
	CIN 2 & 3 treatment costs	564,776	199,475	247,908
	CIN Persistent 2 & 3 treatment costs	3,067,301	435,141	710,367
	Genital warts treatment costs	3,094,714	3,094,714	335,580
	Cervical cancer treatment costs	1,087,169	96,483	157,511
				
Life-years (LY)		1,692,651	1,695,651	1,695,338
QALYs		1,717,089	1,720,571	1,720,299
				
DISCOUNTED				
Total costs		6,880,106	15,034,926	14,210,399
Life-years (LY)		711,164	711,800	711,734
QALYs		735,991	736,775	736,784

*In addition to screening

From the cost-benefit analysis, the incremental savings is $8.23 m with the quadrivalent vaccine and $9.47 m with the bivalent vaccine, or an advantage of $1.24 m for the bivalent over the quadrivalent vaccine. For the cost-effectiveness analysis, the cost per life year saved for the quadrivalent vaccine compared to no vaccine was $12,866, compared to $12,827 for the bivalent vaccine. Comparing the bivalent to the quadrivalent vaccine, the ICER is $12,488, showing that the bivalent vaccine saves more lives for the cost. However, the cost per QALY saved for the quadrivalent vaccine compared to no vaccine was $9,071, while it was $10,392 for the bivalent vaccine, with the quadrivalent vaccine dominating the bivalent vaccine due to the additional QALY effect from reduction in genital warts.

### Sensitivity analysis

For the sensitivity analyses, we compared 3 different outcomes - the quadrivalent vaccine to screening alone, the bivalent vaccine to screening alone and the bivalent vaccine to the quadrivalent vaccine. Table 4 shows selected sensitivity analyses results. Decreasing vaccine coverage while keeping vaccination costs constant (assuming that vaccine purchases are sunk costs) increased the ICER and decreases cost-benefit across the board, especially at vaccine coverage levels ≤40% where vaccination is not cost-effective. If vaccination costs can be capped to only those who receive vaccination, then either vaccine remains cost effective even at 20% vaccination levels.

**Table 4:** Sensitivity analysis for the imput parameters on ICER and Cost-Benefit

Parameters	ICER (SGD/LY saved), discounted			ICER (SGD/QALY saved), discounted			Cost Benefit Analysis (million SGD), discounted		
	Quadrivalent vaccine vs No vaccine	Bivalent vaccine vs No vaccine	Bivalent vaccine vs Quadrivalent vaccine	Quadrivalent vaccine vs No vaccine	Bivalent vaccine vs No vaccine	Bivalent vaccine vs Quadrivalent vaccine	Quadrivalent vaccine vs No vaccine	Bivalent vaccine vs No vaccine	Bivalent vaccine vs Quadrivalent vaccine
Base case	12,866	12,827	12,488	9,071	10,392	Dom	8.23	9.47	1.24
									
Vaccine coverage (base:100%) - assuming vaccine purchase costs for the cohort are sunk costs									
20%	86,397	83,454	56,178	61,804	67,631	Dom	-6.25	-6.57	-0.33
40%	40,444	39,322	29,109	28,847	31,864	Dom	-2.72	-2.75	-0.03
60%	25,124	24,607	19,981	17,861	19,938	Dom	0.92	1.21	0.29
80%	17,464	17,246	15,337	12,367	13,973	Dom	4.67	5.23	0.63
									
Vaccine coverage (base:100%) - assuming vaccine costs are as consumed									
20%	11,524	15,873	56,178	8,059	12,818	Dom	1.75	1.43	-0.33
40%	12,804	14,417	29,109	9,010	11,668	Dom	3.38	3.25	-0.03
60%	13,034	13,733	19,981	9,166	10,727	Dom	4,92	5.21	0.29
80%	13,003	13,241	15,337	9,185	11,122	Dom	6,67	7.3	0.63
									
Vaccine efficacy (Bivalent base: 88.3%, Quadrivalent base: 79.4%)									
70%	15,008	17,237	Dom	10,369	13,894	Dom	6.43	5.31	-1.11
90%	10,863	12,326	Dom	7,733	9,932	Dom	11.13	10.17	-0.96
									
Vaccination costs (base: SGD$400 for both)									
Decrease to SGD$300 for both	8,521	8,942	12,572	5,922	7,205	Dom	10.96	11.96	1
Decrease to SGD$200 for both,	4,109	4,988	12,572	2,775	4,019	Dom	13.46	14.46	1
Decrease to SGD$100 for both	Dom	1,034	12,572	Dom	833	Dom	15.96	16.96	1
									
Booster (base: none)									
5 years	91,149	83,707	19,486	67,830	70,487	Dom	-36.07	-35.52	0.54
10 years	47,045	43,778	15,584	36,192	38,134	Dom	-10.94	-10.14	0.8
20 years	27,321	26,048	15,067	19,483	21,093	Dom	0.3	1.13	0.84
									
Screening coverage rate (base: 17%)									
10%	12,424	12,398	12,171	9,120	10,409	Dom	9.2	10.29	1.08
25%	13,670	13,558	12,593	9,199	10,552	Dom	7.68	8.63	0.95
40%	15,200	14,845	11,783	9,628	10,977	Dom	6.43	7.35	0.92
Effectiveness against viral warts									
70%	13,348	12,827	8,996	9,665	10,392	57,447	8.23	9.46	1.23
80%	13,143	12,827	10,768	9,366	10,392	640,633	8.35	9.46	1.11
100%	12,721	12,827	14,408	8,777	10,392	Dom	8.59	9.46	0.86

Dom = Dominated by quadrivalent vaccine

Lowering the overall vaccine efficacy increases ICER and decreases cost-benefit for both vaccination strategies compared to no vaccination, but vaccination still remains cost-effective even if no cross-reactivity against low-risk HPV types is present. In the latter scenario, the quadrivalent vaccine dominates the bivalent vaccine even for the additional cost per life year saved, due to the additional reduction on genital warts.

Decreasing vaccination costs for both vaccines decreases the overall ICER and increases the cost-benefit of the vaccination strategy. Conversely, overall ICER for all comparisons increases and cost-benefit decreases when vaccine waning and administering a booster in the future are considered, although the vaccination strategies are still cost-effective up to 10 yearly boosters.

Changing the cervical cancer screening rates through pap smears affects the outcomes - increasing screening coverage rates increases ICER and decreases cost-benefit for vaccination due to the higher rates of early detection of CIN states which can be treated before they develop cancer. Increasing screening coverage also decreases the cost per life year saved for the bivalent vaccine compared to the quadrivalent vaccine because screening reduces CIN cases and therefore the advantage that the quadrivalent vaccine has in reducing the number of CIN cases due to non-oncogenic HPV.

The ICER for the quadrivalent vaccine vs. no vaccine increases with reduced effectiveness of the quadrivalent vaccine against genital warts. At 100% effectiveness the quadrivalent vaccine vs. no vaccine is more cost-effective compared to the bivalent vaccine vs. no vaccine across all 3 measures. Changing the sensitivity of the pap- smear tests, the compliance to treatment, or the cost of the interventions does not change the outcome substantially - vaccination remains cost-effective and the bivalent vaccine remains the more cost-beneficial strategy compared to the quadrivalent vaccine, and more-cost effective per life year saved. However, when using cost-per QALY saved, the quadrivalent vaccine dominates the bivalent vaccine across the board due to the additional QALYs saved from reducing genital warts.

**Figure 3:**
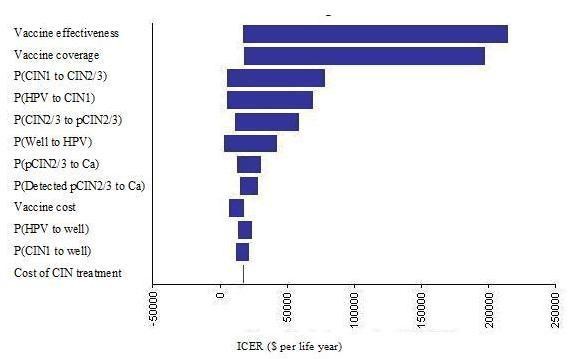
One-way sensitivity analysis tornado diagrams for the quadrivalent vaccine

**Figure 4:**
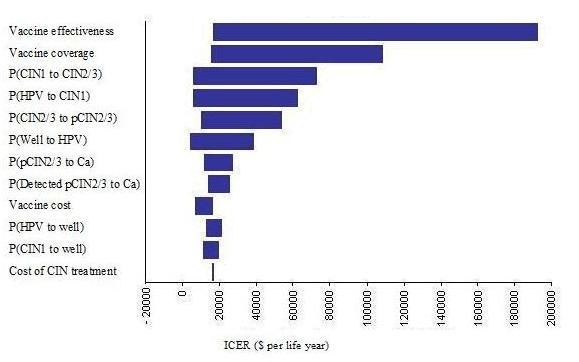
One-way sensitivity analysis tornado diagrams for the bivalent vaccine

The results of the one-way sensitivity analysis on ICER are shown in Figures 3 and 4. It is evident that the primary outcome is most affected by the vaccine effectiveness and the percentage of vaccine coverage. This means that vaccine parameters are the most important factors in the overall outcomes. The probability of infection with HPV, the probability of conversion from HPV to CIN1, and the transition probabilities between the CIN states and back to the well state also have some impact on the outcome. This is likely due to the importance of these parameters on the number of CIN cases that require treatment, and the eventual number of cancer cases. This also means that prevention of HPV infection and CIN is important to reduce the impact from the disease.

## Discussion

During the last few decades, cervical cytology programmes have demonstrated their usefulness in reducing cervical cancer mortality and morbidity in many developed countries. However, it is a well-established fact that to be successful in population-based cervical screening, the coverage of the population screened must be adequate and this has often proved difficult to achieve. Although the incidence has declined over the years, cervical cancer, which is a preventable cancer, is still a common gynecological cancer in Singapore. With the global arrival of HPV vaccines, vaccination may be a successful complement to screening and decision on adoption of HPV vaccination will need to be made across individual countries.

The results of this study conclusively shows that HPV vaccination is a cost effective strategy at the population level, as the ICERs across most vaccination strategies were much lower than the per-capita GDP for Singapore, and the vaccine strategies were more cost-beneficial under a wide range of circumstances. This is similar to a recent study in Taiwan which showed the cost-effectiveness of universal vaccination of adolescent girls [51].

As a base case, the cost per life year saved for the bivalent vaccine was slightly lower compared to the quadrivalent vaccine, and the cost-benefit higher. At the same time, the cost per QALY saved is slightly lower for the quadrivalent vaccine compared to the bivalent vaccine. This is because the quadrivalent vaccine resulted in benefits from reduction in genital warts and CIN, but the bivalent vaccine increased life-years saved. Policy makers will therefore have to consider these factors (higher reduction in QALY versus more lives saved) in choosing between the 2 vaccines, but either option is much better than no vaccination at all.

The bivalent vaccine is most cost-beneficial at higher vaccine coverage rates of >40%, while the quadrivalent vaccine is more cost-effective and cost-beneficial at lower coverage rates as the number of lives saved are reduced. The quadrivalent vaccine also dominates the bivalent vaccine in the absence of cross-reactivity against non-16/18 oncogenic HPV types, due to the additional reduction on genital warts. This is similar to another study in Ireland which shows that the quadrivalent vaccine was more cost-effective assuming both vaccines have similarly high protective efficacy against HPV [29].

Of interest, if vaccine purchases are a priori and are sunk costs, the ICER increases substantially for both vaccines as vaccine coverage decreases, and cost-benefit decreases. Under this assumption, vaccine coverage of ≤40% would render vaccination not cost-effectiveness as an overall strategy. It is therefore important for policy makers rolling out universal vaccination strategies with stockpiled vaccines to ensure sufficient vaccine uptake to maintain high societal economic benefit, or to purchase vaccines on an as required basis based on vaccination uptake.

In addition, the waning of immunity and additional vaccinations contribute substantial to the vaccination costs. Boosters at intervals of once every 20 years still maintain the cost-effectiveness of vaccination. However, more frequent boosters of less than 10 years render vaccination a non-cost-effective strategy. Waning of immunity results in costs far greater compared to the absence of cross-protection to non-16/18 oncogenic HPV types or to genital warts. It is therefore more important for vaccine manufacturers to increase the efficacy of their vaccines in terms of long-term immunity.

This study does not take into account the dynamics of viral transmission and thus underestimating the impact of herd immunity. Transmission models that accounted for herd immunity suggest that vaccination and screening strategy would be much more attractive compared to screening-only - Chesson et al [9] further demonstrated that herd immunity could reduce ICER by 37.9%. In addition, women who adhere to previous cervical screening tests may have better compliance with subsequent tests, but with the lack of information in the local context, we did not perform individual-based modelling. Also, the model does not take into consideration the potential reduction of other HPV-related cancers like adenocarcinoma of the cervix, vulvar carcinoma or laryngeal papillomatosis. Indirect costs such as absence of work or transportation costs for patients with cervical cancer are also excluded. Given that this study did not include herd immunity effects, we did not study the outcomes of vaccinating boys or optimal catch-up strategies, and we did not consider catch up vaccination, which could increase the immediate benefits of vaccination programs.

## Conclusions

We demonstrate that vaccination of adolescent girls, in addition to current cytology-based screening, is a cost-effective use of healthcare resources. The bivalent vaccine saves more lives compared to the quadrivalent vaccine, while the quadrivalent vaccine has lower cost per QALYs saved compared to the bivalent vaccine. The main advantage of vaccination will be to reduce cervical cancer mortality but the full benefits of vaccination will be observed 10-20 years after its introduction. Further studies should focus on quantifying the duration of vaccine protection, and use dynamic models to examine the efficiencies of different screening and vaccine strategies in reducing HPV-related disease. Studies should also focus on optimal synergies between screening and vaccination and on affordability and equity in delivery.

## Competing interests

This study was funded by a research grant from GSK Biologicals. VJL, SKT, and TMY have received research funding from GSK.

## Authors' contributions

VJL and TMY conceived the study, collected the data, performed the analysis, and wrote the first draft of the manuscript together. SKT conceived the study, collected the data, and wrote the manuscript. YLT conceived the study, collected the data, and wrote the manuscript. All authors have read and approved the final manuscript.

## Pre-publication history

The pre-publication history for this paper can be accessed here:

http://www.biomedcentral.com/1471-2458/11/203/prepub
